# Safety and Tolerability of Letetresgene Autoleucel (GSK3377794): Pilot Studies in Patients with Advanced Non–Small Cell Lung Cancer

**DOI:** 10.1158/1078-0432.CCR-24-1591

**Published:** 2024-11-22

**Authors:** Mehmet Altan, Gilberto Lopes, T. Jeroen N. Hiltermann, Ramaswamy Govindan, Liza C. Villaruz, Emiliano Calvo, Martin J. Edelman, Muhammad Furqan, Joel Neal, Enriqueta Felip, Jennifer W. Carlisle, John V. Heymach, Róisín Eilish O’Cearbhaill, Marjorie Zauderer, Michael Chisamore, Ellie Corigliano, Ioanna Eleftheriadou, Stefan Zajic, Ben Jenkins, Sophia Goodison, Sunil Suchindran, Natalia Ramos-Hernandez, Nidale Tarek, Adam J. Schoenfeld

**Affiliations:** 1 Thoracic/Head and Neck Medical Oncology, MD Anderson Cancer Center, Houston, Texas.; 2 University of Miami Health System, Miami, Florida.; 3 University of Groningen, University Medical Center Groningen, Groningen, the Netherlands.; 4 Washington University School of Medicine in St. Louis, St. Louis, Missouri.; 5 UPMC Hillman Cancer Center, Pittsburgh, Pennsylvania.; 6 START Madrid-CIOCC, Centro Integral Oncologico Clara Campal, Madrid, Spain.; 7 Fox Chase Cancer Center, Philadelphia, Pennsylvania.; 8 Carver College of Medicine, University of Iowa, Iowa City, Iowa.; 9 Stanford Cancer Institute, Stanford University, Palo Alto, California.; 10 Vall d’Hebron University Hospital and Vall d’Hebron Institute of Oncology (VHIO), Barcelona, Spain.; 11 Winship Cancer Institute, Emory University School of Medicine, Atlanta, Georgia.; 12 Memorial Sloan Kettering Cancer Center, New York City, New York.; 13 Merck & Co., Inc., Rahway, New Jersey.; 14 GSK, Collegeville, Pennsylvania.

## Abstract

**Purpose:**

The study aims to evaluate the safety, tolerability, and antitumor response of letetresgene autoleucel (lete-cel), genetically modified autologous T cells expressing a T-cell receptor specific for New York esophageal squamous cell carcinoma 1 (NY-ESO-1)/LAGE-1a shared epitope, alone or in combination with pembrolizumab, in HLA-A*02–positive (HLA-A*02:01, HLA-A*02:05, and/or HLA-A*02:06) patients with NY-ESO-1– and/or LAGE-1a–positive non–small cell lung cancer.

**Patients and Methods:**

Study 208749 was a single-arm study of lete-cel alone. Study 208471 was a multiarm study of lete-cel alone or in combination with pembrolizumab in patients with advanced or recurrent non–small cell lung cancer.

**Results:**

More than 2,500 patients were screened for target expression. In the multiarm study, 738 (45%) of 1,638 tested patients were HLA-A*02–positive. NY-ESO-1 and LAGE-1a testing was positive in 12% (62/525) and 4% (15/348) of tested patients, respectively. Forty-one patients positive for HLA-A*02 and antigen expression were screened in the single-arm study. Overall, 43 patients underwent leukapheresis and 18 received lete-cel across studies. Lete-cel demonstrated a manageable safety profile. No fatal treatment-related serious adverse events (AE) were reported in either study. Cytopenias and cytokine release syndrome were the most common treatment-emergent AEs. Combining pembrolizumab with lete-cel did not seem to increase toxicity over lete-cel alone. Limited antitumor activity was observed; one of 18 patients had a durable response persisting for 18 months. Pharmacokinetic data showed similar T-cell expansion in all patients.

**Conclusions:**

Extensive HLA-A*02 and antigen expression testing was performed to identify potential participants. Lete-cel was generally well tolerated and had no unexpected AEs. Antitumor activity was observed in a limited number of patients.

Translational Relevance

Letetresgene autoleucel (lete-cel; GSK3377794) is a genetically modified autologous T cell that expresses a T-cell receptor recognizing a New York esophageal squamous cell carcinoma 1/LAGE-1a shared epitope. The efficacy of T-cell therapy targeting New York esophageal squamous cell carcinoma 1 has been demonstrated in previous clinical trials in hematologic malignancies and solid tumors. Based on efficacy demonstrated in other tumor types, lete-cel was evaluated in two pilot studies, alone or in combination with pembrolizumab, in patients with non–small cell lung cancer. To date, this is the largest experience with a T-cell receptor therapy in non–small cell lung cancer. Overall, our data highlight a manageable safety profile, some antitumor activity, and complexities associated with the design and implementation of HLA-restricted T cell–based therapies. The screening efforts and enrollment challenges in this large-scale study helped develop a unique understanding of the landscape of HLA typing and cancer testis antigen expression in lung cancer, which can be applied to future HLA-based T-cell therapy trial design.

## Introduction

The use of immunotherapy with immune checkpoint inhibitors (ICI) targeting the PD-1/PD-L1 axis alone or in combination with anticytotoxic T lymphocyte–associated protein 4, with or without chemotherapy, has been associated with improved outcomes compared with chemotherapy alone in non–small cell lung cancer (NSCLC; refs. 1–6). Despite these improvements, primary or acquired resistance to ICIs remains a clinical challenge (7). Targeted therapies specific to oncogenic driver mutations, such as *EGFR*, anaplastic lymphoma kinase (*ALK*), and *ROS1*, have substantially enhanced outcomes compared with chemotherapy in a subset of patients (8, 9), but the development of resistance to targeted therapy represents another challenge. ICIs are not effective in patients with certain tumor genomic aberrations such as activating *EGFR* mutations, *ALK*, and *ROS1* translocations, and patients with these tumors are commonly excluded from immunotherapy trials owing to their lack of responsiveness to checkpoint blockade (10).

Strategies aimed at establishing *de novo* antitumor immunity or rescuing native antitumor immunity via delivery of engineered T cell–based cellular therapies have demonstrated promise in multiple solid tumors, including in lung cancer (11–16). T-cell receptor (TCR)–based therapy uses genetically engineered lymphocytes to target antigens derived from tumor-specific proteins [e.g., cancer testis antigens (CTA) and mutated tumor antigens, such as KRAS] presented by HLA molecules on the tumor cell surface as “non-self neoantigens” and can generate a T-cell immune response (17). The treatment process with TCR-based therapies involves patient screening for HLA typing and target tumor-specific protein, leukapheresis, generation of TCR-transduced T cells, lymphodepletion, and infusion of the TCR-transduced T cells (18). The CTA New York esophageal squamous cell carcinoma 1 (NY-ESO-1)/LAGE-1a are appealing targets because of their known immunogenicity and their upregulated expression on certain tumors while maintaining restricted expression on normal tissue. The NY-ESO-1 protein has been shown to be generally homologous to LAGE-1a (approximately 84% shared protein sequence identity; refs. 19–22). Efficacy of T-cell therapy targeting NY-ESO-1 has been demonstrated in previous clinical trials in hematologic malignancies (e.g., multiple myeloma), as well as in patients with synovial sarcoma and melanoma (23–30).

Letetresgene autoleucel (lete-cel; GSK3377794) is an affinity-enhanced T-cell therapy that consists of autologous CD4^+^ and CD8^+^ T cells genetically modified to express a TCR that recognizes the NY-ESO-1/LAGE-1a shared epitope, *SLLMWITQC*, upon presentation on HLA-A*02:01, HLA-A*02:05, or HLA-A*02:06 (24, 26). Lete-cel has shown significant activity in synovial sarcomas, an immunologically “cold” tumor in which NY-ESO-1 is upregulated at a high level (28). NY-ESO-1 and LAGE-1a are also upregulated in both driver oncogene-positive and negative NSCLC, making them potential targets for lete-cel in patients with NSCLC (31).

Based on the efficacy of adoptive T-cell transfer with lete-cel demonstrated in other tumor types, we conducted two pilot studies to evaluate the safety, tolerability, and efficacy of lete-cel alone (study 208749, NCT02588612) and in combination with pembrolizumab (study 208471, NCT03709706) in patients with advanced NSCLC. To date, these are the largest clinical studies of a TCR therapy in NSCLC.

## Patients and Methods

### Overview of study design and primary objectives

Study 208749 is a single-arm, open-label pilot study evaluating the safety and clinical activity of lete-cel in patients with stage IIIB or IV, or recurrent, NSCLC, whereas study 208471 is a multiarm, open-label, phase Ib/IIa study evaluating the safety and clinical activity of lete-cel alone (arm A) or in combination with pembrolizumab (arms B and C) in patients with stage IIIB or IV, or recurrent, NSCLC (32). The original sponsor for the single-arm study was Adaptimmune (ADP-0011-004), with sponsorship transferring to GSK prior to study closure. Both studies complied with the ethical principles of the Declaration of Helsinki. An overview of each study design is shown in Supplementary Fig. S1; a high-level comparison between the two studies is shown in Table 1. Key inclusion and exclusion criteria for the single-arm and multiarm studies are shown in Supplementary Table S1A and S1B, respectively. Eligible patients were ≥18 years of age, diagnosed with advanced NSCLC (stage IIIB or IV), were HLA-A*02–positive (HLA-A*02:01, HLA-A*02:05, and/or HLA-A*02:06), and had tumors expressing NY-ESO-1 or LAGE-1a evaluated on tumor tissue from a formalin-fixed, paraffin-embedded archival sample or fresh biopsy.

**Table 1.** Key comparison overview of single- and multiarm lete-cel NSCLC trials.

	Single-arm study	Multiarm study
Key study details	Pilot, open-label trial of lete-cel monotherapy	Phase Ib/IIa, open-label trial of lete-cel ± pembrolizumab
Patient population	Advanced/recurrent NSCLC, HLA-A*02:01, HLA-A*02:05, and HLA-A*02:06–restricted, tumor positive for NY-ESO-1 and/or LAGE-1a	
Key endpoints	Primary: safety Secondary: ORR, TTR, DOR, DCR, PFS	Primary: safety/ORR Secondary (efficacy): TTR, DOR, DCR, PFS Secondary (pharmacokinetics): *C*_max_, *T*_max_, AUC
Treatment arms	One treatment arm: lete-cel monotherapy With or without actionable genetic aberrations	Three treatment arms: No actionable genetic aberrations, per NCCN: A – lete-cel monotherapy B – lete-cel + pembrolizumab Actionable genetic aberrations, per NCCN: C – lete-cel + pembrolizumab
Target sample size	10	45
HLA-A*02 and NY-ESO-1/LAGE-1a screening	Separate prescreening protocol (ADP-0000-001, NCT02636855)	As part of multiarm study
Cell dose	1–8 × 10^9^ transduced T cells• One patient received 0.84 × 10^9^ transduced T cells due to low yield (waiver)	1–15 × 10^9^ transduced T cells
Manufacturing	First dose: Adaptimmune pilot supply Second dose: GSK pilot supply	GSK pilot and intended commercial supply

Abbreviations: AUC, area under the concentration–time curve; DCR, disease control rate; *T*_max_, time of peak plasma concentration; TTR, time to recurrence.

The primary objective of the single-arm study was to evaluate the safety and tolerability of lete-cel in the selected patient population. For the multiarm study, the primary objectives were to evaluate the safety, tolerability, and efficacy of lete-cel in patients who received treatment alone or in combination with pembrolizumab (Table 1).

Study protocols and patient informed consent documentation were approved by institutional review boards. Written informed consent was required from all study patients prior to study initiation.

### Patient enrollment and screening methods

The initial target enrollment for the single-arm monotherapy study was 10 patients; patients were pre-screened for the relevant HLA alleles (HLA-A*2:01, HLA-A*2:05, and/or HLA-A*02:06) and target antigen expression (NY-ESO-1/LAGE-1a) through a separate prescreening protocol, ADP-0000-001 (NCT02636855) before entering the screening phase of the protocol to determine eligibility for enrollment (Supplementary Fig. S2). Eligibility criteria were grouped into two parts and eligibility screening involved the following two steps: leukapheresis eligibility screening and lymphodepletion eligibility screening. Patients were planned to receive lete-cel monotherapy as a single intravenous infusion. Patients who had a confirmed response and documented disease progression, and whose tumors continued to express NY-ESO-1 and/or LAGE-1a, were considered for a second lete-cel infusion.

The initial target enrollment for the multiarm immunotherapy study was 45 dosed patients, or 15 per arm. Eligibility criteria were grouped into four parts and eligibility screening involved the following four steps: target expression screening, leukapheresis eligibility screening, lymphodepletion eligibility screening, and pembrolizumab eligibility screening. Patients who did not have actionable genetic aberrations per National Comprehensive Cancer Network (NCCN) guidelines (33) were assigned to receive either lete-cel monotherapy as a single intravenous infusion (arm A) or a single intravenous infusion of lete-cel on day 1 followed by pembrolizumab to be initiated on day 22 (arm B; Supplementary Fig. S3), with patients being assigned to arm A first and then to arm B. Any patients in arm A who progressed within 25 weeks following lete-cel infusion had the option of receiving pembrolizumab following a benefit–risk evaluation. Patients with actionable genetic aberrations (e.g., sensitizing *EGFR* mutations or *ALK* translocations) per NCCN guidelines were assigned to arm C and received the same treatment as patients in arm B. There was no re-treatment with lete-cel in this study.

### Target expression screening

For target expression screening, HLA type was evaluated using DNA extracted from a blood sample, and archival or fresh tumor tissue was tested for NY-ESO-1 and LAGE-1a expression.

In the multiarm study, the consented patient population was screened using an investigational-use only IHC clinical trial assay for NY-ESO-1 protein expression using the E978 anti-NY-ESO-1 mAb, and an investigational-use only RT-PCR clinical trial assay for LAGE-1a RNA expression detection.

NY-ESO-1 IHC–positive tumor expression was defined as *P* score ≥ 10%, 1+, 2+, and 3+, and LAGE-1a RNA–positive tumor expression was defined as cut-off = 4 dCT and below. LAGE-1a testing was done in reflex to NY-ESO-1 IHC–negative results (<10%, 1+, 2+, and 3+ IHC; *P* score). Tumor samples submitted for testing included archival specimens or fresh biopsies from either a primary or metastatic lesion. Calculations for NY-ESO-1 and LAGE-1a prevalence excluded repeat testing or nonunique specimens.

### Leukapheresis, lymphodepletion, and lete-cel infusion

For both studies, patients who met all eligibility criteria following screening were enrolled and underwent leukapheresis to obtain autologous cells for the manufacture of lete-cel (NY-ESO-1^c259^ TCR T-cell investigational product; ref. 29). In the single-arm study, lete-cel was manufactured via pilot manufacturing supply, whereas in the multiarm study, lete-cel was manufactured via either pilot or intended commercial manufacturing supply. The pilot lete-cel manufacturing process has been previously described (29); the intended commercial supply followed a similar manufacturing process.

Lete-cel was administered to patients following lymphodepleting chemotherapy with cyclophosphamide and fludarabine (27, 34). A range of 1 to 8 × 10^9^ transduced T cells for the single-arm study and 1 to 15 × 10^9^ transduced T cells for the multiarm study was administered by a single intravenous infusion on day 1 for both studies. The cell dose range was selected based on manufacturing capabilities (35–37). For both studies, planned lymphodepletion consisted of fludarabine 30 mg/m^2^/day at days −8, −7, −6, and −5 and cyclophosphamide 900 mg/m^2^/day intravenously at days −7, −6, and −5; adjusted for age ≥60 years, renal impairment, increased body weight, or risk of prolonged cytopenia, among others. Refer to study protocols for more information. Lymphodepletion could be given as outpatient treatment at the discretion of the investigator. Patients received mesna (sodium 2-mercaptoethane sulfonate) per institutional guidelines and G-CSF starting 24 hours following the last dose of chemotherapy until neutrophil count recovery.

### Pembrolizumab dosing (multiarm study)

Patients in arms B and C of the multiarm study received pembrolizumab at the approved dose of 200 mg every 3 weeks. The first administration was on day 22 after administration of lete-cel and then every 3 weeks for up to 35 cycles or until disease progression.

### Study endpoints

In both studies, the primary safety endpoints included assessment of adverse events (AE). In the multiarm study, the primary efficacy endpoint was investigator-assessed objective response rate (ORR), per RECIST v1.1.

Secondary efficacy endpoints in both studies included time to response, duration of response (DOR), disease control rate, and progression-free survival (PFS). In addition, the single-arm study also evaluated investigator-assessed ORR (per RECIST v1.1) as a secondary endpoint. Exploratory endpoints for both studies included the correlation between lete-cel persistence and response. Both studies also evaluated overall survival (OS) as an exploratory endpoint.

### Study assessments

Following lete-cel infusion, patients were monitored closely and received appropriate supportive care measures. Patients routinely underwent physical exams, safety evaluations, medical imaging, and biological sample collection until the end of interventional phase criteria were met or until patient withdrawal. AEs of special interest (AESI) reported in both studies included cytokine release syndrome (CRS), immune effector cell–associated neurotoxicity syndrome (ICANS), pancytopenia/aplastic anemia, graft-versus-host disease, Guillain–Barre syndrome, and pneumonitis. In the multiarm study, AESIs of grade 4 neutropenia lasting ≥28 days and treatment-related inflammatory response at tumor sites were also reported; in addition, all single-cell cytopenias were listed based on reported laboratory results. Refer to study protocols for more information.

The end of the interventional phase was defined as completion of the follow-up period in the interventional phase, disease progression per RECIST v1.1 in the single-arm study or confirmed disease progression per immune-related RECIST (iRECIST) in the multiarm study, or death after lete-cel infusion, whichever comes first.

On completion of the interventional phase or early withdrawal, patients were transitioned to a long-term follow-up (LTFU) study for observation of delayed AEs for 15 years after lete-cel infusion, in accordance with the FDA and the European Medicines Agency requirements for gene therapy clinical trials.

### Lete-cel persistence and pharmacokinetics

Patient peripheral blood mononuclear cells (PBMC) were collected for monitoring the persistence of lete-cel–modified cells. DNA PCR-based methods were used to detect the *Psi* gene contained in the lentiviral vector used to transduce the T cells. Persistence, defined by the duration of maximum serum concentration (*C*_max_; vector copies/µg of genomic DNA), was derived as time from T-cell infusion until *C*_max_ was no longer detectable.

### Antidrug antibodies, replication-competent lentivirus, and integration site analysis

Serum samples for determination of antibodies against lete-cel were taken from all participants for antidrug antibody testing.

Replication-competent lentivirus (RCL) was monitored using a PCR-based assay that detects and measures copies of the gene coding for the vector’s envelope protein, vesicular stomatitis virus G protein, which is necessary for the assembly of pseudotyped infectious lentiviral particles but is absent from the vector’s backbone. Vesicular stomatitis virus G protein quantitative PCR testing was carried out on lentiviral vector, T-cell product, and PBMCs at timepoints indicated in the schedule of assessments for each study protocol.

At the first instance of >1% of PBMCs testing positive for the *Psi* gene at or after 1-year post-infusion, the participant’s PBMCs were evaluated for integration site analysis by assessing clonality and the possibility of insertional oncogenesis following regulatory recommendations (38, 39).

### Statistical analysis

For the single-arm study, the sample size was based on clinical judgment; enrollment of 10 participants was planned. The small study was not powered to conduct any statistical hypothesis testing on either primary or secondary endpoints. For the multiarm study, the sample size was determined using a Bayesian predictive adaptive design that allowed the study to be monitored more frequently while maintaining the desired type I error and power. The sample size was based on testing the hypothesis for each arm separately and not for a comparison between arms. Enrollment of approximately 18 participants was planned for each arm to ensure approximately 15 evaluable participants would receive lete-cel infusion within the target dose range.

The intention-to-treat (ITT) population included all patients who enrolled in the trial and met all eligibility criteria, and the modified ITT (mITT) population comprised patients who received lete-cel infusions. The mITT2 population included all patients who received a second lete-cel infusion.

Safety analyses were performed using the ITT and mITT populations for treatment-dependent safety analysis. For both studies, AEs (coded by Medical Dictionary for Regulatory Activity terms) were tabulated by System Organ Class and Preferred Term and were further classified by toxicity (using the NCI Common Terminology Criteria for Adverse Events, v4.03).

In both studies, the population used for the efficacy analysis was the mITT population. Tumor assessments for response and progression were evaluated according to RECIST v1.1 (40) and iRECIST (41). Any ORR endpoint was summarized by two-sided 95% confidence intervals (CI) using exact Clopper–Pearson methods. DOR (in the subset of patients with a confirmed response), PFS, and OS were summarized and displayed graphically using Kaplan–Meier methodology to estimate the median, 25th and 75th percentiles, and two-sided 95% CIs. Descriptive statistics were provided for disposition, demographics, and safety.

Final analyses for each study were conducted when all patients who received lete-cel infusions (mITT) moved to the separate LTFU protocol, declined LTFU, were lost to follow-up, withdrew early, or died. No interim analyses were planned for the single-arm study. Separate interim analyses were planned for each arm in the multiarm study after at least 10 patients were available in that arm. No formal statistical hypotheses were tested in either study.

### Data availability

The results summary for this study is available on clinicaltrials.gov, the default register for GSK Human Subject Research. All datasets analyzed in the article will be available upon request to the editors and peer reviewers, and to the community at the time of publication. For interventional studies that evaluate our medicines, patient-level data will be made available to independent researchers, subject to review by an independent panel, at www.clinicalstudydatarequest.com. To protect the privacy of patients and individuals involved in our studies, GSK does not publicly disclose patient-level data.

## Results

### Screening and patient identification

For the single-arm study, patients were confirmed as positive for HLA-A*02 and antigen expression under a separate prescreening protocol from Adaptimmune (ADP-0000-001, NCT02636855). More than 1,000 patients were pre-screened; these data are not included in the current article. Forty-one of the patients identified as positive for HLA-A*02 and antigen expression under ADP-0000-001 consented to the single-arm study to pursue screening (Supplementary Fig. S2).

**Figure 1.**
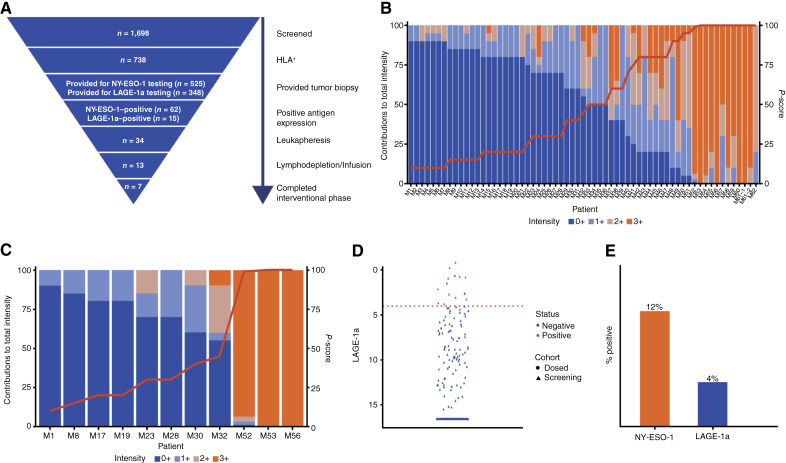
Patient attrition and NY-ESO-1 and LAGE-1a expression profiles in the multiarm study. **A,** Patient attrition for multiarm study. A total of 1,698 patients in the multiarm study were screened and signed the informed consent form. Out of 13 dosed patients, six withdrew prior to meeting interventional phase completion criteria, per protocol. **B,** NY-ESO-1 IHC *P*-score distribution of screened NY-ESO-1–positive patients in the multiarm study. NY-ESO-1–positive protein expression was detected as a continuous variable with distribution between 10% and 100% expression. There were 62 patients whose tumors expressed NY-ESO-1 with a *P* score of ≥10%, 1+, 2+, and 3+, with each vertical bar representing a unique tumor sample depicted by contributing IHC intensity of 0+ in blue, 1+ in light blue, 2+ in light orange, and 3+ in orange (425 patients whose tumors expressed NY-ESO-1 <10%, 1+, 2+, and 3+ are not displayed). One patient (ID #61 in the figure) had two samples. **C,** NY-ESO-1 expression profile of dosed NY-ESO-1–positive patients in the multiarm study. The mITT population based on NY-ESO-1 IHC expression is individually represented in the vertical bars with the *P*-score broken down by contributing IHC intensity of 0+ in blue, 1+ in light blue, 2+ in light orange, and 3+ in orange. The patients are ordered by *P* score, which is indicated by the red line. NY-ESO-1–negative, LAGE-1a–positive patients in the mITT population not shown. **D,** LAGE-1a RNA expression distribution of all tested patients in the multiarm study. The plot of patients tested for LAGE-1a RT-PCR RNA expression, in which the red line indicates the cutoff = 4 ΔCT, with positive cases depicted in red and negative cases depicted in blue. Triangles represent all patients screened. The two patients dosed who were LAGE-1a–positive are distinguished in the figure with a red circle. Patients with “no expression” were imputed as having the lowest observable value divided by 3. The vertical axis is reversed (zero is higher); lower LAGE-1a score measured by RT-PCR represents higher expression levels. **E,** NY-ESO-1 and LAGE-1a prevalence. This is a bar chart displaying NY-ESO-1 and LAGE-1a prevalence. LAGE-1a testing was performed on tissue samples in reflex to NY-ESO-1–negative status for when tissue was available. Prevalence includes unique samples from the same patient. NE, not evaluable.

A total of 1,698 patients in the multiarm study signed the screening informed consent. Of those, 1,638 patients were tested for HLA-A*02 and 738 (45%) patients were HLA-A*02–positive. Among the 525 patients who provided tissue for NY-ESO-1 testing, 62 (12%) were positive [samples from 38 (7%) patients were not evaluable]. Three hundred forty-eight patients were tested for LAGE-1a; 15 (4%) were positive [samples from six (2%) patients were not evaluable; Fig. 1A; Supplementary Fig. S3].

### Patient characteristics (mITT population), apheresis, and treatment

Individual patient demographics and characteristics of both mITT populations, including tumor stage and histology, antigen expression, lymphodepletion regimen, and lete-cel treatment, are shown in Table 2. Patient representativeness by sex, age, race/ethnicity, and geography is reported in Supplementary Table S2.

**Table 2.** Patient characteristics: tumor stage and histology, prior therapy, LDR, and lete-cel treatment (mITT population).

Patient	Age, years/sex	Disease stage at diagnosis/histology	Actionable mutation^a^	No. of prior lines of therapy	Prior ICI	HLA-A*02 status	NY-ESO-1/LAGE-1a (NY-ESO-1 *P* score)	Transduced T-cell dose, × 10^9^	LDR dose^b^, mg/m^2^ (days)	Best response
Single-arm study (*n* = 5)										
S1	64/F	IV/adenocarcinoma	*EGFR*	2	No	02:06 heterozygous	NY-ESO-1 (30%)	2.2	Flu: 30 (×3) Cy: 600 (×3)	PD
S2	29/F	IV/adenocarcinoma	—	5	Yes	02:01 homozygous	NY-ESO-1 (30%)	5.0	Flu: 30 (×4) Cy: 900 (×3)	PD
S3	70/M	IV/adenocarcinoma	—	3	Yes	02:01 heterozygous	NY-ESO-1 (40%)	0.8	Flu: 30 (×3) Cy: 600 (×3)	PD
S4	52/M	IV/adenocarcinoma	NA	1	No	02:01 heterozygous	NY-ESO-1 (40%)	3.7	Flu: 20 (×3) Cy: 600 (×3)	SD
S5First dose/second dose	59/M	IV/adenocarcinoma	—	4	Yes	02:01 heterozygous	NY-ESO-1 (90%/100%)	3.0/3.0	Flu: 30 (×3) Cy: 600 (×3) Flu: 30 (×3) Cy: 600 (×3)	PR/SD
Multiarm study (*n* = 13)										
M53	64/M	IV/adenocarcinoma	—	1	Yes	02:01 heterozygous	NY-ESO-1 (100%)	5.2	Flu: 30 (×2) Cy: 600 (×2)	SD
M32	68/M	IV/adenocarcinoma	—	2	Yes	02:01 homozygous	NY-ESO-1 (45%)	3.8	Flu: 20 (×3) Cy: 600 (×3)	SD
M63	77/M	Unknown/adenocarcinoma	—	1	Yes	02:05 heterozygous	LAGE-1a	7.8	Flu: 20 (×3) Cy: 600 (×3)	SD
M64	49/M	IV/adenocarcinoma	—	3	Yes	02:01 heterozygous	NY-ESO-1 (100%)	8.0	Flu: 30 (×4) Cy: 900 (×3)	PD
M19	62/M	IV/adenocarcinoma	—	2	Yes	02:01 heterozygous	NY-ESO-1 (20%)	3.8	Flu: 30 (×3) Cy: 600 (×3)	SD
M65	74/F	III/adenocarcinoma	—	3	Yes	02:01 heterozygous	LAGE-1a	4.3	Flu: 22.5 (×3) Cy: 600 (×3)	SD
M52	61/F	IV/adenocarcinoma	—	2	Yes	02:01 heterozygous	NY-ESO-1 (99%)	5.3	Flu: 20 (×3) Cy: 600 (×3)	SD
M1	50/F	IV/adenocarcinoma	*EGFR*	2	Yes	02:01 heterozygous	NY-ESO-1 (10%)	7.4	Flu: 20 (×4) Cy: 900 (×3)	PD
M23	65/F	IV/adenocarcinoma	*ALK*	4	No	02:01 heterozygous	NY-ESO-1 (30%)	8.0^c^	Flu: 20 (×3) Cy: 600 (×3)	SD
M17	61/F	IV/adenocarcinoma	*EGFR*	3	No	02:01/02:06 heterozygous	NY-ESO-1 (20%)	3.0	Flu: 20 (×3) Cy: 600 (×3)	PD
M8	76/F	IV/Adenocarcinoma	*EGFR*	2	Yes	02:06 heterozygous	NY-ESO-1 (15%)	3.9	Flu: 30 (×2) Cy: 600 (×2)	NE
M28	56/M	IV/adenocarcinoma	*ALK*	3	No	02:01 heterozygous	NY-ESO-1 (30%)	1.0	Flu: 30 (×4) Cy: 900 (×3)	PD
M30	54/F	IV/adenocarcinoma	*EGFR*	2	No	02:01 heterozygous	NY-ESO-1 (40%)	8.1	Flu: 30 (×4) Cy: 900 (×3)	SD

Abbreviations: Cy, cyclophosphamide; Flu, fludarabine; LDR, lymphodepleting regimen; NA, not available; NE, not evaluable; PD, progressive disease; SD, stable disease.

a For the single-arm study, considered actionable mutations were *ROS1*, *EGFR*, and *ALK*.

b Standard LDR of Flu: 30 mg/m^2^ (4 days) and Cy: 900 mg/m^2^ (3 days). Participants underwent LDR dose modifications due to various reasons including: ≥60 years of age, renal impairment, increased body weight, or risk of prolonged cytopenia. Refer to study protocols for more information.

c One of the four transduced T-cell bags for this participant was not fully infused due to an infusion-related reaction; therefore, they received anywhere between 6 and 8 × 10^9^ transduced cells.

In the single-arm study, most of the patients in the mITT population were White (80%) and male (60%), with a median age of 59.0 years (range, 29–70 years). Four (80%) patients were HLA-A*02:01–positive, whereas one (20%) was found to be HLA-A*02:06–positive (Table 2). All had NY-ESO-1–positive tumors. In the multiarm study, most patients in the mITT population were White (92%) and female (54%), with a median age of 62.0 years (range, 49–77 years). Eleven (85%) patients were HLA-A*02:01–positive, two (15%) patients were HLA-A*02:06–positive (frequencies include one patient who was positive for both HLA-A*02:01 and HLA-A*02:06), and one (8%) patient was HLA-A*02:05–positive (Table 2). Eleven (85%) patients had NY-ESO-1–positive tumors, and two (15%) patients had LAGE-1a–positive tumors.

Dosed patients across studies received standard-of-care anticancer therapy prior to receiving lete-cel. This included targeted therapy for patients with actionable genetic aberrations, following NCCN or equivalent country-level guidelines (Table 2). In arm C of the multiarm study, four patients had actionable *EGFR* mutations and two had actionable *ALK* mutations (Table 2).

In the single-arm study, nine patients underwent leukapheresis, of whom five patients underwent lymphodepletion and T-cell infusion (Supplementary Fig. S2). Reasons for withdrawal prior to lymphodepletion were stable disease at the time of T-cell expiry, withdrawal at investigator’s decision, and low cell yield at apheresis. The median number of transduced T cells was 3.02 × 10^9^ (range, 0.84–4.98). One patient received a second lymphodepleting treatment and T-cell infusion following progression after the first lete-cel treatment. In the multiarm study, a total of 34 patients underwent leukapheresis, of whom 13 underwent lymphodepletion and lete-cel infusion (Fig. 1A; Supplementary Fig. S3). Seven and six patients were assigned to arms A and C, respectively, according to their genetic aberration status (Table 1). Arm B of the trial was never opened. Contributing to attrition rates among patients who underwent leukapheresis in the multiarm study were failed eligibility, lack of fitness, death due to underlying disease, withdrawal at patient/investigator discretion, and study closure. The median number of transduced T cells was 5.2 × 10^9^ (range, 1.0–8.1). Seven (54%) patients (three in arm A; four in arm C) received at least one infusion of pembrolizumab. Two patients in arm C did not receive pembrolizumab (one due to ongoing AEs and one due to disease progression and death). Administration of pembrolizumab was delayed in two patients in arm C due to AEs, including one who did not receive any doses at the time of disease progression). T-cell products meeting the protocol-defined minimum requirement of 1 × 10^9^ transduced T cells were successfully manufactured for 33 out of 34 patients who underwent leukapheresis in the multiarm study; one patient died in between apheresis and manufacturing, and thus, manufacturing never started. Of the 34 patients who underwent leukapheresis, four underwent repeat leukapheresis and nine required a second T-cell manufacturing process. Manufacturing data are not available for the single-arm study.

All dosed patients across studies were followed until completion of the interventional phase or withdrawal. The multiarm study was terminated early at the discretion of the sponsor without reaching criteria for interim analyses or the target of 15 dosed patients per treatment arm.

### Target expression screening and analysis

The NY-ESO-1 IHC assay in the multiarm study produced predominantly cytoplasmic and nuclear staining across the dynamic range of staining intensities (0+, 1+, 2+, and 3+; ref. 42). The expression profile distribution spanned from being fully negative (100%, 0+) to 100% of tumor cells stained, revealing a wide range of expression in a continuous variable fashion, as determined by *P* score (maximum of 100%; Fig. 1B). The NY*-*ESO-1 expression profile for the mITT population in the multiarm study is depicted in Fig. 1C and shows the distribution of both the *P* score and the relative contribution of staining intensity. Antigen analysis of tumor samples that were reflexed to LAGE-1a testing also revealed a wide distribution of LAGE-1a scores (Fig. 1D). The prevalence of NY-ESO-1 and LAGE-1a positivity was assessed by including unique specimens for all patients and excluding multiple tests of the same tissue block, resulting in 12% and 4% positivity, respectively (Fig. 1E). Nonevaluable samples were also included.

### Safety data

There were no fatal treatment-emergent AEs (TEAE) reported in both studies. In the single-arm study, cytopenias were the most common total and grade ≥3 TEAEs (Supplementary Table S3A). In the multiarm study, cytopenias and CRS were the most common TEAEs, and cytopenias were the most common grade ≥3 TEAEs (Supplementary Table S3B). In the single-arm study, four patients (80%) reported at least one serious TEAE [two patients (40%) experienced serious TEAEs of grade ≥3; Table 3]. In the multiarm study, eight patients across arms (62%) reported at least one serious TEAE (all were grade ≥3; Table 4). There were no serious TEAEs attributed to pembrolizumab.

**Table 3.** Safety results: grade ≥3 and total AESI^a^ and SAE. Single-arm study (mITT population).

Treatment-emergent AESI, *n* (%)	Grade ≥3	Total^b^
Any event	1 (20)	3 (60)
CRS	1 (20)	2 (40)
Hematopoietic cytopenias		
Pancytopenia	0	1 (20)
ICANS		
Encephalopathy	0	1 (20)

Treatment-emergent SAE, *n* (%)	Grade ≥3	Total^b^
Any event	2 (40)	4 (80)
CRS	1 (20)	2 (40)
Pneumonia	1 (20)	2 (40)
Anemia/red blood cell count decreased	1 (20)	1 (20)
Hypotension	1 (20)	1 (20)
Nausea	1 (20)	1 (20)
Rash/maculopapular rash	1 (20)	1 (20)
Respiratory distress	1 (20)	1 (20)
Constipation	0	1 (20)
Encephalopathy	0	1 (20)
Hemorrhage intracranial	0	1 (20)

Abbreviations: AESI, adverse event of special interest; CRS, cytokine release syndrome; GBS, Guillain–Barre syndrome; GvHD, graft-vs.-host disease; ICANS, immune effector cell‐associated neurotoxicity syndrome; mITT, modified intention-to-treat; SAE, serious adverse event.

a AESIs included CRS, ICANS, pancytopenia/aplastic anemia, GvHD, GBS, and pneumonitis.

b Total includes participants with events across all grades.

**Table 4.** Safety results: grade ≥3 and total AESI^a^ and SAE. Multiarm study (mITT population).

	Arm A (*n* = 7)		Arm C (*n* = 6)	
Treatment-emergent AESI, *n* (%)	Grade ≥3	Total^b^	Grade ≥3	Total^b^
Any event	5 (71)	6 (86)	4 (67)	6 (100)
CRS	0	5 (71)	0	4 (67)
Hematopoietic cytopenias^c^				
Leukopenia/white blood cell decreased	5 (71)	5 (71)	1 (17)	1 (17)
Neutropenia/neutrophil count decreased	4 (57)	5 (71)	3 (50)	4 (67)
Thrombocytopenia/platelet count decreased	3 (43)	5 (71)	1 (17)	2 (33)
Lymphopenia/lymphocyte count decreased	2 (29)	3 (43)	1 (17)	1 (17)
Anemia/red blood cell count decreased	1 (14)	2 (29)	2 (33)	4 (67)
Hemoglobin decreased	1 (14)	1 (14)	0	0
Pancytopenia	1 (14)	2 (29)	0	0
ICANS	1 (14)	2 (29)	0	0

Treatment-emergent SAEs, *n* (%)	Grade ≥3	Total^b^
Arm A (*n* = 7)		
Any event	5 (71)	5 (71)
CRS	0	1 (14)
Empyema	1 (14)	1 (14)
ICANS	1 (14)	1 (14)
Nausea	1 (14)	1 (14)
Pancytopenia	1 (14)	1 (14)
Pericardial effusion	1 (14)	1 (14)
Toxic skin eruption	1 (14)	1 (14)
Urinary tract infection	1 (14)	1 (14)
Vomiting	1 (14)	1 (14)
Arm C (*n* = 6)		
Any event	3 (50)	3 (50)
CRS	0	1 (17)
Acute kidney injury	1 (17)	1 (17)
Aspiration	1 (17)	1 (17)
Blood bilirubin increased	1 (17)	1 (17)
Fatigue	1 (17)	1 (17)
Infusion-related reaction	1 (17)	1 (17)
Rash/maculopapular rash	1 (17)	1 (17)
Small intestinal obstruction	1 (17)	1 (17)

Abbreviations: AESI, adverse event of special interest; CRS, cytokine release syndrome; GBS, Guillain–Barre syndrome; GvHD, graft-vs.-host disease; ICANS, immune effector cell-associated neurotoxicity syndrome; mITT, modified intention-to-treat; SAE, serious adverse event.

a AESIs included CRS, ICANS, pancytopenia/aplastic anemia, GvHD, GBS, and pneumonitis. AESIs of grade 4 neutropenia lasting ≥28 days and treatment-related inflammatory response at tumor sites were also assessed in the multiarm study.

b Total includes participants with events across all grades.

c Table listing AESIs of pancytopenia combined with single-cell cytopenia based on reported laboratory results.

AESIs (defined in footnotes of Tables 3 and 4) were reported in three (60%) patients in the single-arm study: two serious AEs (SAE) of CRS, one SAE of ICANS, and one non-SAE of pancytopenia. The two CRS events from the single-arm study were grades 1 and 3 and lasted from days 9 to 17 and 4 to 11 after T-cell infusion, respectively; tocilizumab was administered for the grade 3 event (Table 3). AESIs were reported in 12 (92%) patients in the multiarm study: nine AEs of CRS (two serious), two AEs of ICANS (one serious), and two AEs of pancytopenia (one serious; Table 4). The events of CRS in the multiarm study had an onset of 1 to 8 days following administration of lete-cel and a median duration of 5 days. The two serious events of CRS were both grade 2 and lasted from days 8 to 12 and 7 to 12 after T-cell infusion, respectively. The latter of the two serious events of CRS was treated with tocilizumab and corticosteroids. There were no reports of graft-versus-host disease or Guillain–Barre syndrome in either study. In both studies, all AESIs were considered related to study treatment, and all were reported as resolved.

### Clinical efficacy data

**Figure 2.**
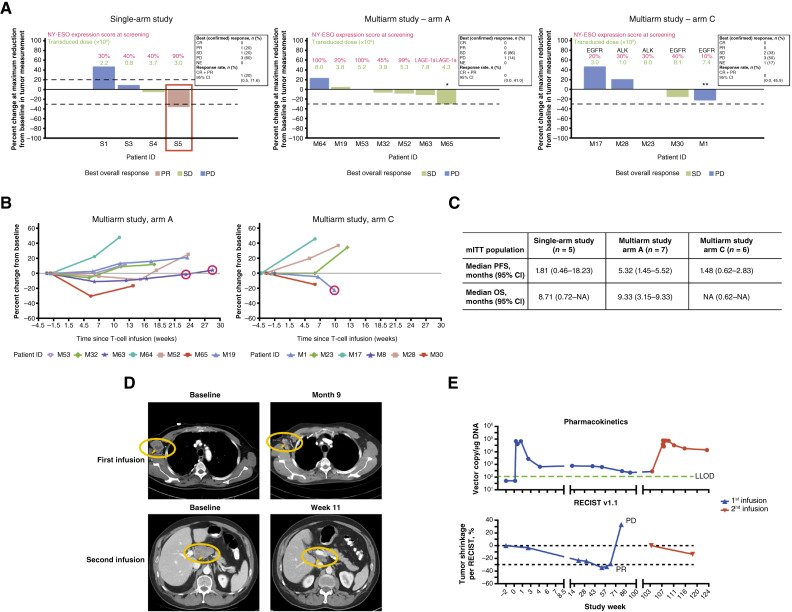
Patient tumor responses in the single- and multiarm studies. **A,** Best overall response in the single- and multiarm studies (confirmed). In the single-arm study, data following first infusion only. One patient did not have measurable target lesions at baseline and does not appear in this plot; one patient received a transduced cell dose below the required dose level, per protocol, due to waiver granted. In the multiarm study, *arm A: one patient (M65) had unconfirmed PR. **Arm C: one patient (M1) had unequivocal progression due to new lesion and one patient died prior to first post-treatment scan and does not appear in this plot. **B,** Percent change from baseline in target lesion diameter in the multiarm study. Arm A patients are shown at left, and arm C is shown at right. Red circles indicate new target lesions. **C,** Median PFS and OS for each study. **D,** Radiographic images of case study patient S5, single-arm study. One patient from the single-arm study (S5) received two separate infusions, achieving best overall responses of confirmed PR from first infusion and SD from the second, per RECIST 1.1. The top left image is baseline before the first infusion, and top right is after 9 months. The bottom left image is baseline before the second infusion, and the bottom right is at week 11 after second infusion. **E,** Peak persistence and tumor shrinkage of case study patient S5, single-arm study. CR, complete response; LLOD, lower limit of detection; NA, not available; NE, not evaluable; PD, progressive disease; SD, stable disease.

In the single-arm study, one of the five patients (ORR 20%; 95% CI, 0.5–71.6) had a confirmed partial response (PR) following the first T-cell infusion. The remaining patients had stable disease (one patient) and progressive disease (three patients) as best response (Fig. 2A). In the multiarm study, six (86%) and two (33%) patients had stable disease as best response in arms A and C, respectively, and no patients had a PR or complete response (Fig. 2A and B).

In the single-arm study, four patients (80%) experienced disease progression per RECIST 1.1 and one (20%) had death from progression not confirmed by RECIST 1.1. In the multiarm study, 10 patients (77%) across arms [six (86%) patients in arm A and four patients (67%) in arm C] experienced disease progression per RECIST 1.1. One death was reported in arm C due to complications of underlying disease. Two patients withdrew from the study. The median PFS in the single-arm study was 1.81 months (95% CI, 0.46–18.23), whereas median PFS was 5.32 months (95% CI, 1.45–5.52) in arm A and 1.48 months (95% CI, 0.62–2.83) in arm C of the multiarm study. Median OS was 8.71 months (95% CI, 0.72–NA) in the single-arm study and 9.33 months (95% CI, 3.15–9.33) in arm A and not reached (95% CI, 0.62–NA) in arm C of the multiarm study (Fig. 2C).

The patient who responded in the single-arm study (Table 2 patient S5) was heterozygous for HLA-A*02:01 and had a poorly differentiated adenocarcinoma without *EGFR*, *ALK*, *or ROS1* pretreatment alterations, or any other targetable oncogenic mutations. Patient S5 was previously treated with three lines of chemotherapy and immunotherapy (nivolumab) and received pemetrexed as a bridging therapy. The patient had a small reduction in lymphadenopathy that lasted 3 months following prior immunotherapy treatment. Radiographic images illustrating PR per RECIST 1.1 following lete-cel are shown in Fig. 2D. Response was durable until 18 months after initial infusion (Fig. 2E). Following disease progression, the patient was considered to have completed the interventional portion of the study and underwent tumor resection as part of standard of care. The patient received a second lymphodepleting regimen and T-cell infusion approximately 28 weeks after documented progressive disease after first infusion (and 22 weeks after tumor resection). Tumor assessment on initial 8-week scan after second infusion showed stable disease per RECIST 1.1 (with complete response in non-target lesions). The patient then withdrew consent prior to further disease evaluation. Therefore, no DOR data were collected following the second T-cell infusion.

All five patients who completed lymphodepletion and lete-cel infusion in the single-arm study completed the interventional phase, according to protocol definition (RECIST 1.1; Supplementary Fig. S2). Of the 13 patients who completed lymphodepletion and lete-cel infusion in the multiarm study, seven patients completed the interventional phase according to protocol definition (iRECIST; Supplementary Fig. S3). Six patients discontinued the interventional phase early, including four who withdrew prior to scan confirming disease progression per iRECIST (Supplementary Fig. S3).

### Pharmacokinetic data and persistence

**Figure 3.**
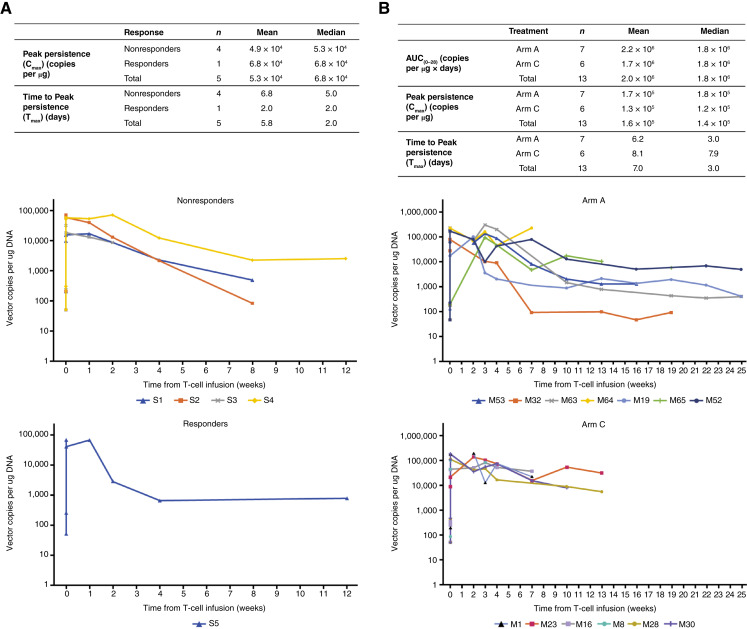
Cellular kinetics profile by patient in the single- and multiarm studies. **A,** Single-arm study. Cellular kinetics profile at first infusion by patient and responder status truncated at 12 weeks (available data), mITT population. **B,** Multiarm study. Cellular kinetics profile by patient and treatment arm truncated at 6 months (first infusion), mITT population. AUC_0–28 days,_ AUC from 0 to 28 days.

In the single-arm study, peak cell persistence (defined as median *C*_max_) was 6.8 × 10^4^ copies per µg in the responder, with shorter median time to *C*_max_ (*T*_max_) in the responder versus non-responders (Fig. 3A). In the multiarm study, mean peak cell persistence was 1.7 × 10^5^ copies per µg in arm A (lete-cel monotherapy), with a median *T*_max_ of 3.0 days. Mean peak persistence was 1.3 × 10^5^ copies per µg in arm C (lete-cel and pembrolizumab combination therapy), with median *T*_max_ of 7.9 days. Mean AUC from 0 to 28 days was 2.2 × 10^6^ copies per µg × days in arm A and 1.7 × 10^6^ copies per µg × days in arm C (Fig. 3B).

### Biomarker data

Two patients in the single-arm study were tested for RCL; both were negative. No integration site analysis was performed because there were no *Psi* DNA copies of PBMCs >1% at 1-year post-treatment. In the multiarm study, all patients were tested for anti-GSK3377794 antibodies at least once and were negative at all timepoints during the study. All patients were tested for RCL at least once and were negative at all timepoints during the study. No patients remained in the study 1-year post-treatment.

## Discussion

We have reported the findings from the 208749 single-arm, open-label pilot study evaluating lete-cel alone and the 208471 multiarm, open-label, phase Ib/IIa study evaluating lete-cel, either alone or in combination with pembrolizumab, in advanced NSCLC. The rationale behind the combination of lete-cel and pembrolizumab in arms B and C of the 208471 multiarm study was to overcome the hostile immunosuppressive microenvironment observed in solid tumors and to augment responses following lete-cel infusion. This rationale is supported by studies demonstrating the synergistic effects of combining NY-ESO-1–specific T cells with anti–PD-1 therapy (43). To date, these are the largest clinical studies of a TCR therapy in NSCLC. Overall, lete-cel was generally well tolerated and had a manageable safety profile consistent with that described in prior and ongoing studies across the lete-cel program (27). The combination of lete-cel administration and a PD-1 inhibitor did not seem to increase toxicity in NSCLC. Antitumor activity was observed for lete-cel in a subset of patients with only one durable response observed among 18 patients treated across studies. Pharmacokinetic data showed similar T-cell expansion between all patients and a comparable T-cell expansion profile to what has been described in prior lete-cel trials (28, 29).

In these studies, large-scale HLA typing and target expression testing of more than 2,500 were required to ultimately treat 18 patients. The extensive screening needed and the high attrition rate experienced during the screening process are important to consider, especially in the context of the rapid growth of HLA-restricted vaccines and T cell–based therapeutics in solid tumors. Stepwise HLA blood and NY-ESO-1/LAGE-1a tumor testing and apheresis can lead to long time periods between initial screening and treatment. Efforts to reduce screening barriers, such as incorporating HLA typing into standard next-generation sequencing platforms, may be critical to screening efficiency and may enable more equitable access to HLA-restricted therapies in the future. Differential HLA-A*02 allele prevalence by race or ethnicity could affect patient access to T cell–based therapies in certain populations (44, 45). Furthermore, timing apheresis can be challenging in patients heavily pretreated with therapies and can typically impair T-cell activity (46, 47). Planning and consideration of cellular therapy earlier in the course of treatment may be beneficial to both patient and T-cell fitness.

Trafficking of T cells and effective infiltration of the tumor are essential for the direct antitumor activity of adoptive T-cell therapy. Poor infiltration of transduced T cells into NY-ESO-1–expressing tissue could have contributed to the limited antitumor activity observed. Locoregional delivery of cell therapies, including direct intrapleural/tumoral injection, is an active area of investigation to overcome this obstacle (48, 49). There are several additional potential mechanisms of resistance to T-cell therapy, including the existence of inhibitory ligands in the tumor microenvironment and endogenous TCRs that can exhaust chimeric antigen receptor T cells (50). Other potential factors contributing to resistance are defects in antigen presentation and HLA loss with prior exposure to PD-1 blockade. Screening for HLA loss of heterozygosity refines neoantigen prediction and may have implications for our understanding of resistance mechanisms and immunotherapeutic approaches targeting neoantigens (51).

Unlike chimeric antigen receptor T cells, TCR-based approaches enable the recognition of targets beyond the cell surface to include targets located within any subcellular compartment. The use of TCRs also enables the recognition of low concentrations of intracellular cognate antigens and mimics the natural function of the T cell by recruiting the endogenous signaling molecules and adhering to correct spatial orientation between the T cell and its target (52–54). However, TCR-based adoptive T-cell therapies still rely on appropriate target expression and recognition, and ultimately, the NY-ESO-1/LAGE-1a epitope–HLA complex may not be an optimal therapeutic target in lung cancer. The affinity and stability of the engineered TCR–HLA interaction also affect the T-cell response, and the utilization of novel approaches, including machine-learning models and structure-based designs, may improve the selection of TCRs and potential outcomes in future studies (55, 56).

Unlike some soft tissue sarcomas, NY-ESO-1/LAGE-1a is not commonly expressed in lung cancer. In addition, NY-ESO-1/LAGE-1a expression in NSCLC is relatively heterogeneous across the patient population and within a given tumor sample (42). Target expression screening in the multiarm study suggests NY-ESO-1 prevalence in NSCLC to be approximately 12% (because LAGE-1a testing in the multiarm study was done in reflex, it may not accurately reflect its prevalence in NSCLC). It should be noted the NY-ESO-1 IHC CTA test and LAGE-1a RT-PCR CTA tests are also investigational and the set cut-offs for positive expression can include low and/or heterogeneous antigen levels. Low and/or heterogeneous antigen levels may help explain the often limited and transient clinical benefits. Owing to the small population in this trial, it is difficult to have enough data to modify the clinical testing cut-offs or to adjust assay performance.

However, consistent with the potential importance of NY-ESO-1/LAGE-1a antigen levels, the only patient in the single-arm study who had a PR to lete-cel treatment had very high (90%) NY-ESO-1 expression. Lete-cel may be more effective in tumors in which NY-ESO-1 is highly and uniformly expressed, such as in sarcomas. High levels of NY-ESO-1 expression with homogeneous distribution have been reported in myxoid/round cell liposarcomas and synovial sarcomas, which might be related to the promising results obtained in adoptive cellular immunotherapy trials (26, 57–59).

T-cell expansion and cell persistence were not meaningfully different in these NSCLC studies compared with previous studies in other indications in which efficacy has been observed (e.g., synovial sarcoma and myxoid/round cell liposarcoma; refs. 23–26). This suggests that expansion and persistence were not likely to explain the limited efficacy in these studies.

In addition to NY-ESO-1, other tumor neoantigens, such as mutant *KRAS* G12D and *KRAS* G12V, are being explored as targets for engineered TCR/adoptive T-cell therapy in solid tumors (60, 61). Other antitumor immunotherapy technologies under development include bispecific T-cell engager antibodies comprising two binding domains that link endogenous T cells to engage specific antigen-expressing tumors (62).

In summary, we demonstrated that lete-cel was generally safe in advanced NSCLC with or without pembrolizumab, but antitumor activity was limited and there were various design challenges associated with both studies that necessitated larger-scale screening efforts and limited study feasibility. Throughout these screening efforts, we gained valuable insights into the landscape of HLA typing and CTA expression in lung cancer, which constitute key learnings for designing future trials of HLA-based therapies. Furthermore, the enrollment challenges outlined above can provide critical insights for design and implementation of future HLA-restricted TCR and vaccine-based therapies, which are increasingly being investigated across solid tumors.

## Supplementary Material

Supplementary Figure 1 Supplementary Figure 1. Patient journey

Supplementary Figure 2 Supplementary Figure 2. Single-arm study – Patient disposition

Supplementary Figure 3 Supplementary Figure 3. Multi-arm study – Patient disposition

Supplementary Table 1 Supplementary Table 1. Key eligibility criteria

Supplementary Table 2 Supplementary Table 2. Representativeness of Study Participants

Supplementary Table 3 Supplementary Table 3. Grade ≥3 and total TEAEs
