# Supplementary material for: Safety and Tolerability of Letetresgene Autoleucel (GSK3377794): Pilot Studies in Patients with Advanced Non–Small Cell Lung Cancer
Source: Clin Cancer Res. 2024 Nov 22;31(3):529–42. doi: 10.1158/1078-0432.CCR-24-1591 (PMC11788651; doi:10.1158/1078-0432.CCR-24-1591)
Supplement: Supplementary Figure 1 — Patient journey [file ccr-24-1591_supplementary_figure_1_suppsf1.pdf]

Supplementary Figure 1. Patient journey

Supplementary Figure 1

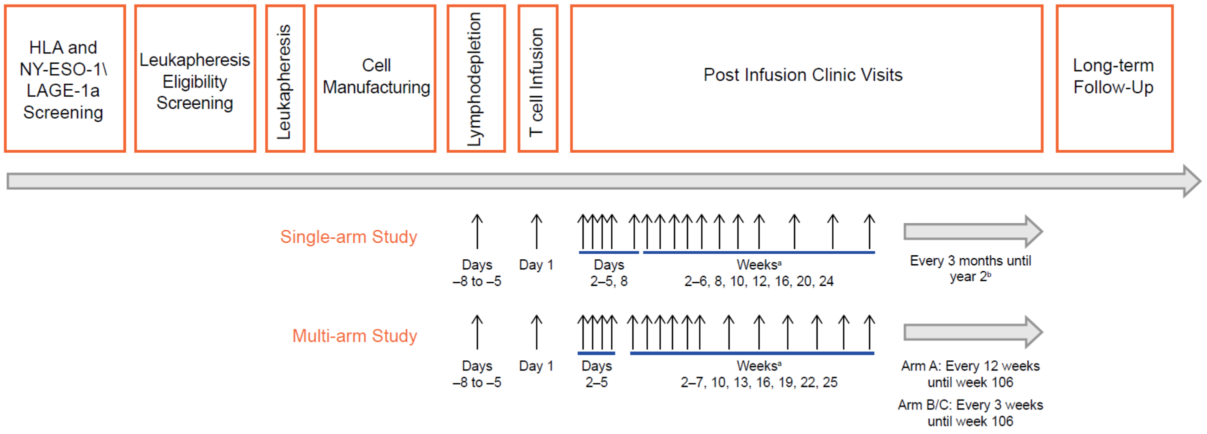

The patient journey in both studies is illustrated as part of the overall study workflow, which involved HLA screening (blood sample) and antigen expression screening for NY-ESO-1\LAGE-1a (tumor sample), leukapheresis eligibility screening, and leukapheresis in eligible patients to supply T cells for GMP-grade cell manufacturing, lymphodepletion, and T-cell infusion. Patients attended post-infusion clinic visits and received long-term follow-up care. Arrows indicate clinic visits.

[a]Single-arm study started at Week 0 and multi-arm study started at Week 1. [b]Second infusion permitted in single-arm study. GMP, good manufacturing practice; HLA, human leukocyte antigen; LAGE-1a, cancer testis antigen 2; NY-ESO-1, New York esophageal squamous cell carcinoma 1.
