# Supplementary material for: Safety and Tolerability of Letetresgene Autoleucel (GSK3377794): Pilot Studies in Patients with Advanced Non–Small Cell Lung Cancer
Source: Clin Cancer Res. 2024 Nov 22;31(3):529–42. doi: 10.1158/1078-0432.CCR-24-1591 (PMC11788651; doi:10.1158/1078-0432.CCR-24-1591)
Supplement: Supplementary Figure 2 — Single-arm study – Patient disposition [file ccr-24-1591_supplementary_figure_2_suppsf2.pdf]

## Supplementary Figure 2. Single-arm study – Patient disposition

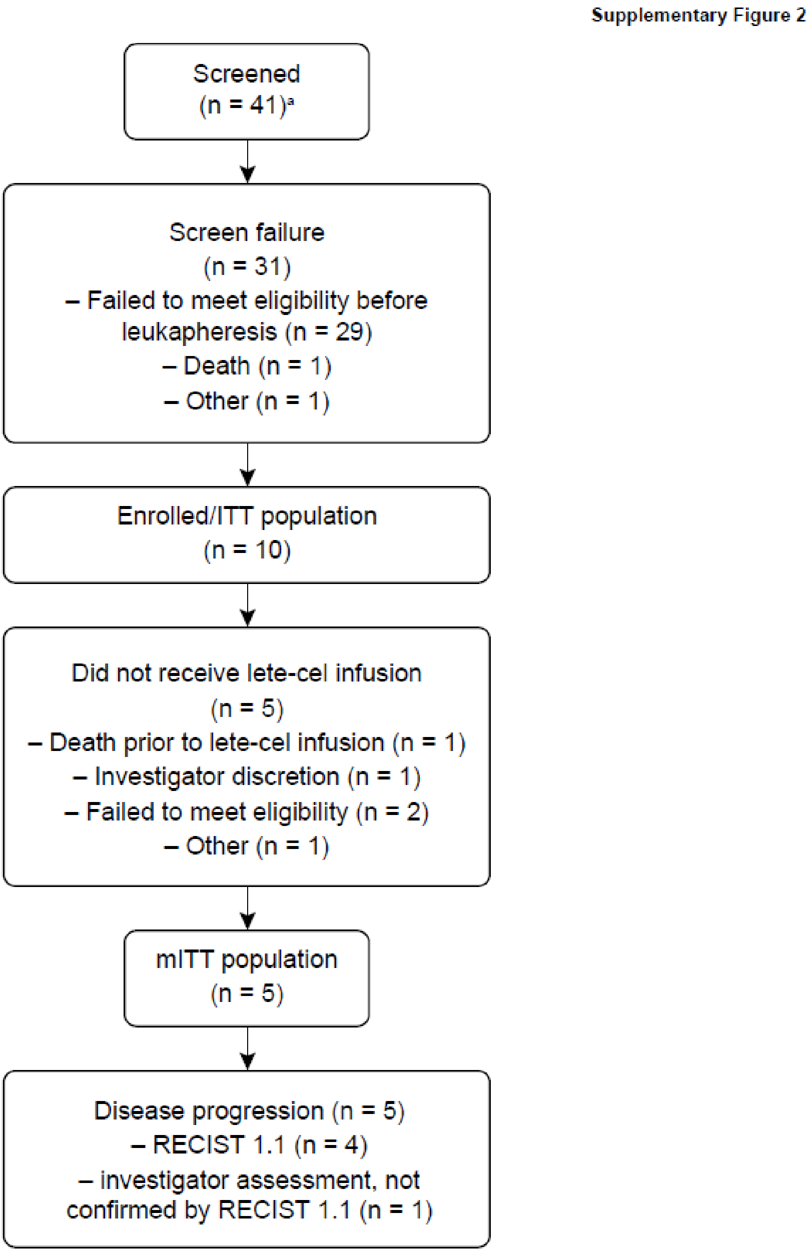


[a]More than 1000 patients were pre-screened under a separate screening protocol from Adaptimmune (ADP-0000-001, NCT02636855) (data not shown). ITT, intention-to-treat; lete-cel, letetresgene autoleucel; mITT, modified intention-to-treat; RECIST, Response Evaluation Criteria in Solid Tumors.
