# Supplementary material for: Safety and Tolerability of Letetresgene Autoleucel (GSK3377794): Pilot Studies in Patients with Advanced Non–Small Cell Lung Cancer
Source: Clin Cancer Res. 2024 Nov 22;31(3):529–42. doi: 10.1158/1078-0432.CCR-24-1591 (PMC11788651; doi:10.1158/1078-0432.CCR-24-1591)
Supplement: Supplementary Figure 3 — Multi-arm study – Patient disposition [file ccr-24-1591_supplementary_figure_3_suppsf3.pdf]

Supplementary Figure 3. Multi-arm study – Patient disposition

Supplementary Figure 3

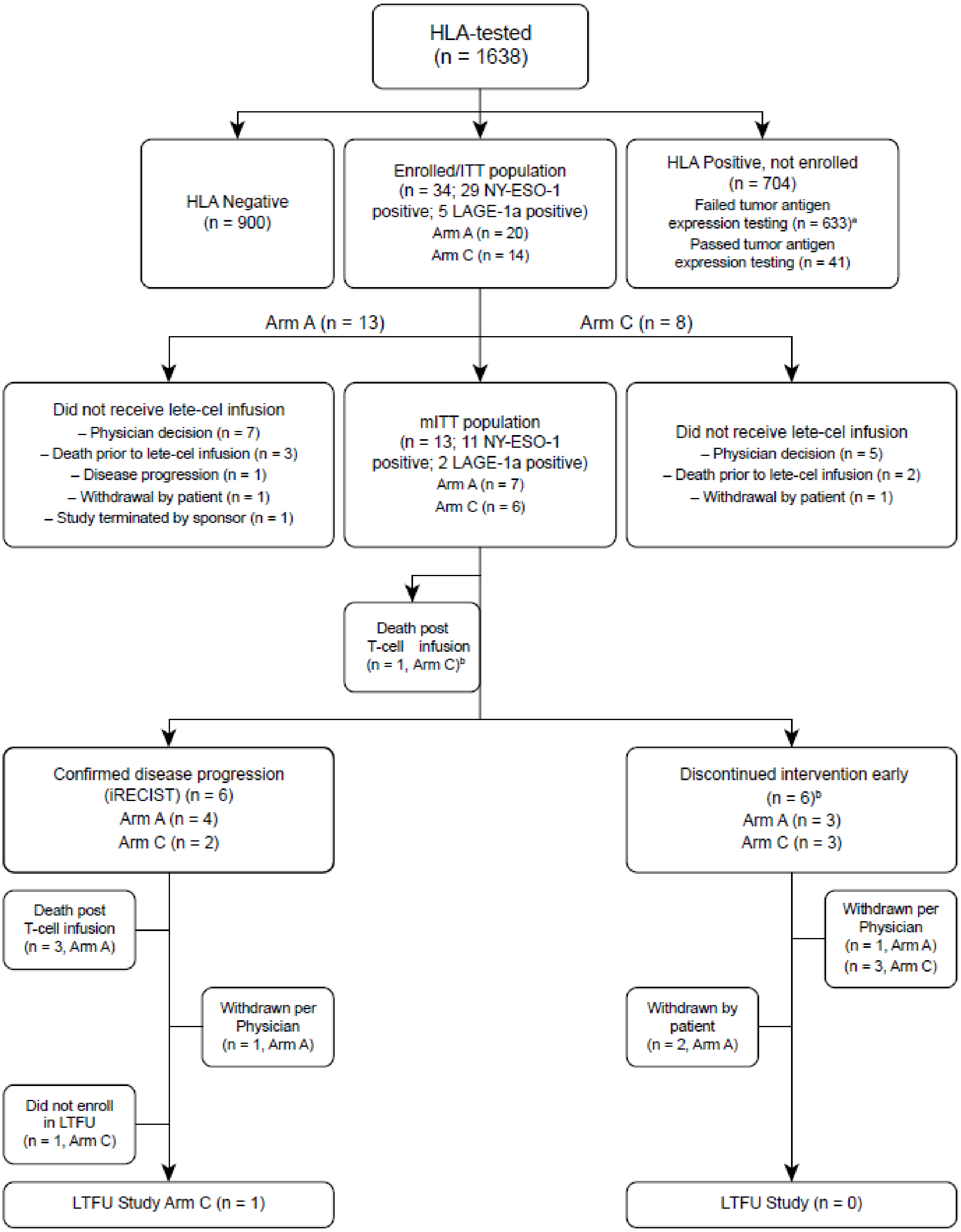

[a]Patients were HLA-positive but were not found to be positive for either NY-ESO-1 or LAGE-1a. [b]Prior to confirmed disease progression (iRECIST) or death or 2 years follow-up post-T-cell infusion. HLA, human leukocyte antigen; ITT, intention-to-treat; LAGE-1a, cancer testis antigen 2; lete-cel, letetresgene autoleucel;

LTFU, long-term follow-up; mITT, modified intention-to-treat; NY-ESO-1, New York esophageal squamous cell carcinoma 1; RECIST, Response Evaluation Criteria in Solid Tumors.
