# Supplementary material for: Safety and Tolerability of Letetresgene Autoleucel (GSK3377794): Pilot Studies in Patients with Advanced Non–Small Cell Lung Cancer
Source: Clin Cancer Res. 2024 Nov 22;31(3):529–42. doi: 10.1158/1078-0432.CCR-24-1591 (PMC11788651; doi:10.1158/1078-0432.CCR-24-1591)
Supplement: Supplementary Table 1 — Key eligibility criteria [file ccr-24-1591_supplementary_table_1_suppst1.pdf]

Supplementary Table 1. Key eligibility criteria

A. Single-arm study

Inclusion criteria	Exclusion criteria
≥18 years of age	Patient had toxicity from previous anti-cancer therapy that had not recovered to grade ≤1 prior to enrollment
HLA-A*02:01, HLA-A*02:05, and/or HLA-A*02:06-positive	
Tumor sample positive for NY-ESO-1 and/or LAGE-1a expression (as reviewed by an Adaptimmune-designated central laboratory)	Patient had CNS metastases except if, on a case-by-case basis after risk–benefit evaluation in consultation with the Sponsor Medical Monitor or designee
Histologically or cytologically confirmed advanced NSCLC (stage IIIB or IV) or recurrent disease	
Patients with known *EGFR* mutations or *ALK* or *ROS1* gene rearrangement must have failed prior TKI therapy	Patient had other active malignancies besides NSCLC within 3 years prior to screening
Measurable disease according to RECIST v1.1 criteria prior to leukapheresis	
Disease progression prior to lymphodepletion chemotherapy	
Patients have failed at least one prior platinum-containing regimen	
ECOG PS 0–1	

ALK, anaplastic lymphoma kinase; CNS, central nervous system; ECOG PS, Eastern Cooperative Oncology Group performance status; EGFR, epidermal growth factor receptor; HLA, human leukocyte antigen; LAGE-1a, cancer testis antigen 2; NSCLC, non-small cell lung cancer; NY-ESO-1, New York esophageal squamous cell carcinoma 1; RECIST, Response Evaluation Criteria in Solid Tumors; TKI, tyrosine kinase inhibitor.

B. Multi-arm study

Inclusion criteria	Exclusion criteria
Target expression screening eligibility criteria	
≥18 years of age<br><br>Histologically or cytologically diagnosed unresectable stage IIIb or stage IV NSCLC<br><br>ECOG PS 0–1<br><br>Tumor tissue sample with associated pathology report is available to perform tumor antigen expression analysis (NY-ESO-1 or LAGE-1a)	Prior treatment:<br><br>a. Previous treatment with genetically engineered NY-ESO-1-specific T cells<br><br>b. Previous NY-ESO-1 vaccine or NY-ESO-1-targeting antibody<br><br>c. Prior gene therapy using an integrating vector<br><br>Prior malignancy other than NSCLC, with the following exceptions:<br><br>Patients with a history of basal cell carcinoma of the skin, superficial bladder cancer, squamous cell carcinoma of the skin, in situ cervical cancer, or have undergone potentially curative therapy with no evidence of that disease recurrence for 5 years since initiation of that therapy
Leukapheresis eligibility screening	
HLA-A*02:01, HLA-A*02:05, and/or HLA-A*02:06-positive<br><br>Tumor reviewed by designated laboratory and confirmed as meeting the pre-defined threshold for expression of NY-ESO-1 and/or, if tested, LAGE-1a	Symptomatic or untreated CNS metastases
Lymphodepletion eligibility screening	
Patients with NSCLC lacking actionable genetic aberrations need to have received at least one line of PD-1/PD-L1 checkpoint blockade therapy<br><br>Patients with NSCLC with actionable genetic aberrations should have received appropriate targeted therapy following NCCN or equivalent country-level guidelines (e.g., ESMO, NICE, etc.)<br><br>Measurable disease per RECIST v1.1, as assessed by local site investigator/radiology<br><br>Clinical and/or radiographic disease progression	

Pembrolizumab eligibility screening	
Radiographically confirmed progressive disease following treatment with lete-cel at or before the scheduled Week 25 scan	Persistence of toxicities, such as CRS ≥grade 2, that preclude treatment with pembrolizumab
Any toxicity must be ≤grade 1 CTCAE (v4.03) at the time of first/dose (except for non-clinically significant toxicities; e.g., alopecia, vitiligo)	

CNS, central nervous system; CRS, cytokine release syndrome; CTCAE, Common Terminology Criteria for Adverse Events; ECOG PS, Eastern Cooperative Oncology Group performance status; ESMO, European Society for Medical Oncology; HLA, human leukocyte antigen; LAGE-1a, cancer testis antigen 2; lete-cel, letetresgene autoleucel; NCCN, National Comprehensive Cancer Network; NICE, National Institute for Health and Care Excellence; NSCLC, non-small cell lung cancer; NY-ESO-1, New York esophageal squamous cell carcinoma 1; PD-1, programmed cell death protein 1; PD-L1, programmed cell death ligand 1; RECIST, Response Evaluation Criteria in Solid Tumors.
