# Supplementary material for: Safety and Tolerability of Letetresgene Autoleucel (GSK3377794): Pilot Studies in Patients with Advanced Non–Small Cell Lung Cancer
Source: Clin Cancer Res. 2024 Nov 22;31(3):529–42. doi: 10.1158/1078-0432.CCR-24-1591 (PMC11788651; doi:10.1158/1078-0432.CCR-24-1591)
Supplement: Supplementary Table 2 — Representativeness of Study Participants [file ccr-24-1591_supplementary_table_2_suppst2.pdf]

Supplementary Table 2. Representativeness of Study Participants

**Cancer type(s)/subtype(s)/stage(s)/condition**	**NSCLC**
**Considerations related to:**	
**Sex**	In a 2021 cross-sectional epidemiological analysis in the United States, the incidence of NSCLC was consistently higher for men than for women. Among 1.28 million new cases of NSCLC recorded during 2010 to 2017 in the United States, 53% were in male individuals (1).
**Age**	In SEER-18, from 2010 to 2017, the median age of diagnosis among all patients with NSCLC in the United States was 70 years of age (1).
**Race/ethnicity**	The SEER-18 database identified 325,138 patients with NSCLC from 2007 to 2018 in the United States. Of these, 75.2% were White (2).
**Geography**	NSCLC occurrence has notable geographical differences due to screening access; incidence and mortality rates for NSCLC are higher in rural versus urban areas. In addition, lower socioeconomic status is also associated with lower survival rates (3).
**Overall representativeness of this study**	In the single-arm study, most patients with NSCLC were male (60%), which is aligned with the literature. In the multi-arm study, the majority of patients were female (54%). The median age of patients (59 [study ID: 208749] and 62 [study ID: 208471] years of age) was slightly lower than the median age reported in the literature. Most patients in this study were White (80% [study ID: 208749] and 92% [study ID: 208471]), which is aligned with the literature. The single-arm study [study ID: 208749] included patients from the United States only. The multi-arm study [study ID: 208471] screened patients in the United Kingdom, the United States, Spain, Canada, and the Netherlands, and treated patients in the United States, the Netherlands, and Spain. Overall, the differences from epidemiological data in the literature may be attributed to the small number of patients in both studies.

NSCLC, non-small cell lung cancer; SEER, Surveillance, Epidemiology, and End Results.

References

1. Ganti AK, Klein AB, Cotarla I, Seal B, Chou E. Update of Incidence, Prevalence, Survival, and Initial Treatment in Patients With Non-Small Cell Lung Cancer in the US. JAMA Oncol 2021;7:1824-32.

2. Primm KM, Zhao H, Hernandez DC, Chang S. Racial and Ethnic Trends and Disparities in NSCLC. JTO Clin Res Rep 2022;3:100374.

3. Kurzrock R, Chaudhuri AA, Feller-Kopman D, Florez N, Gorden J, Wistuba, II. Healthcare disparities, screening, and molecular testing in the changing landscape of non-small cell lung cancer in the United States: a review. Cancer Metastasis Rev 2024.
