# Supplementary material for: Safety and Tolerability of Letetresgene Autoleucel (GSK3377794): Pilot Studies in Patients with Advanced Non–Small Cell Lung Cancer
Source: Clin Cancer Res. 2024 Nov 22;31(3):529–42. doi: 10.1158/1078-0432.CCR-24-1591 (PMC11788651; doi:10.1158/1078-0432.CCR-24-1591)
Supplement: Supplementary Table 3 — Grade ≥3 and total TEAEs [file ccr-24-1591_supplementary_table_3_suppst3.pdf]

Supplementary Table 3. Grade ≥3 and total TEAEs

A. Single-arm study (mITT population)

TEAEs, n (%)	Grade ≥3	Total[a]
Any event	5 (100)	5 (100)
Leukopenia/white blood cell decreased	5 (100)	5 (100)
Lymphopenia/lymphocyte count decreased	5 (100)	5 (100)
Neutropenia/neutrophil count decreased	5 (100)	5 (100)
Anemia/red blood cell count decreased	3 (60)	4 (80)
Thrombocytopenia/platelet count decreased	3 (60)	4 (80)
Nausea	1 (20)	3 (60)
Decreased appetite	0	3 (60)
Fatigue	0	3 (60)
Headache	0	3 (60)
Hypotension	2 (40)	2 (40)
Pneumonia	2 (40)	2 (40)
CRS	1 (20)	2 (40)
Insomnia	1 (20)	2 (40)
Back pain	0	2 (40)
Confusional state	0	2 (40)
Constipation	0	2 (40)
Muscular weakness	0	2 (40)
Oedema peripheral	0	2 (40)
Sinus tachycardia	0	2 (40)

B. Multi-arm study (mITT population)

TEAEs, n (%)	Grade ≥3	Total[a]
**Arm A (n = 7)**		
Any event	6 (86)	7 (100)
Leukopenia/white blood cell decreased	5 (71)	5 (71)
Neutropenia/neutrophil count decreased	3 (43)	5 (71)
Thrombocytopenia/platelet count decreased	3 (43)	5 (71)
Decreased appetite	2 (29)	5 (71)
CRS	0	5 (71)
Alopecia	0	4 (57)
Fatigue	0	4 (57)
Pyrexia	0	4 (57)
Hyponatremia	2 (29)	3 (43)
Lymphopenia/lymphocyte count decreased	2 (29)	3 (43)
Dyspnea	1 (14)	3 (43)
Depression	0	3 (43)
Diarrhea	0	3 (43)
Alanine aminotransferase increased	1 (14)	2 (29)
Anemia/red blood cell count decreased	1 (14)	2 (29)
Blood creatinine increased	1 (14)	2 (29)
Headache	1 (14)	2 (29)
Hypophosphatasemia	1 (14)	2 (29)
ICANS	1 (14)	2 (29)
Nausea	1 (14)	2 (29)
Pancytopenia	1 (14)	2 (29)
Asthenia	0	2 (29)

Cough	0	2 (29)
Dry skin	0	2 (29)
Hallucination	0	2 (29)
Hypokalemia	0	2 (29)
Hypomagnesemia	0	2 (29)
Hypotension	0	2 (29)
Insomnia	0	2 (29)
Rash morbilliform	0	2 (29)
Rash/rash maculo-papular	0	2 (29)
Tumor pain	0	2 (29)
Weight decreased	0	2 (29)
**Arm C (n = 6)**		
Any event	6(100)	6 (100)
Neutropenia/neutrophil count decreased	3 (50)	4 (67)
Anemia/red blood cell count decreased	2 (33)	4 (67)
Rash/rash maculo-papular	2 (33)	4 (67)
CRS	0	4 (67)
Back pain	1 (17)	3 (50)
Decreased appetite	1 (17)	3 (50)
Fatigue	1 (17)	3 (50)
Alopecia	0	3 (50)
Vomiting	0	3 (50)
Alanine aminotransferase increased	1 (17)	2 (33)
Thrombocytopenia/platelet count decreased	1 (17)	2 (33)
Aspartate aminotransferase increased	0	2 (33)
Diarrhea	0	2 (33)
Headache	0	2 (33)

Nausea	0	2 (33)
Oropharyngeal pain	0	2 (33)

Summary of all TEAEs occurring in two or more participants. [a]Total includes participants with events across all grades. CRS, cytokine release syndrome; ICANS, immune effector cell-associated neurotoxicity syndrome; mITT, modified intention-to-treat; TEAE, treatment-emergent adverse event.
